# The multi-omics fallacy in microbiome science

**DOI:** 10.1371/journal.pcbi.1014700

**Published:** 2026-08-25

**Authors:** Rebecca Lewandowski

**Affiliations:** Department of Molecular and Cellular Biology, University of Arizona, Tucson, Arizona, United States of America; University College London, UNITED KINGDOM OF GREAT BRITAIN AND NORTHERN IRELAND

## Abstract

Artificial intelligence and machine-learning-assisted multi-omics have expanded the scale and ambition of microbiome research, but they have also sharpened an older interpretive problem. Biologically plausible structure is too easily mistaken for biological explanation. This Perspective defines the multi-omics fallacy as claim inflation that occurs when integrating microbial, host, environmental, spatial, and clinical data is assumed to move interpretation from association toward verified mechanism without a corresponding gain in measurement, localization, temporal resolution, functional linkage, or perturbation. The risk is not that computational integration lacks value. It is that predictions, imputations, inferred pathways, feature attributions, and cross-layer networks can acquire mechanistic authority before their biological status has been established. In microbiome science, where stool readouts, taxonomic abundance, inferred function, and predicted metabolites often serve as proxies for host-microbial interaction, this slippage can make uncertain claims appear more complete than the evidence allows. Computational confidence can amplify structured artifact when systematic error becomes learnable. This Perspective proposes a model-to-mechanism burden of proof that distinguishes prediction from explanation, imputation from observation, attribution from causality, cross-layer coherence from mechanism, and diagnostic performance from biological validity. This framework is intended to strengthen, not constrain, computational microbiome science by clarifying which outputs support classification or hypothesis generation and which require direct measurement, localization, temporal analysis, functional validation, or perturbation. Used this way, computational models can help expose uncertainty, prioritize experiments, identify fragile claims, and sharpen biological questions. The result would be a more powerful form of computational microbiome science, one in which models do not stand in for mechanisms but guide the work needed to earn them.

## Introduction

Microbiome science has gained computational power faster than interpretive discipline. In this Perspective, artificial intelligence and machine-learning-assisted computational methods refer to data-driven approaches used in omics integration, including classifiers, ordination, dimensionality reduction, imputation, pathway prediction, network inference, feature attribution, and predictive or generative systems. These methods differ in architecture and purpose, but they share a common interpretive risk. Their outputs can be granted biological meaning beyond the evidence that produced them. Building on calls for credible, reproducible, and causally restrained inference as artificial intelligence-assisted approaches expand in microbiome research [1], this Perspective asks how computational outputs should be interpreted before they become biological claims.

The problem begins when computational outputs acquire stronger evidentiary status without additional evidence. A prediction can read as observation, an imputed feature as a measured organism, an inferred pathway as active biology, and feature attribution as host relevance. These outputs become biological explanations only when the relevant organism, metabolite, pathway, exposure, or host response has been measured, localized, functionally linked, or perturbed.

Computational output should direct biological testing rather than replace it. In work on resistance to *Clostridioides difficile*, metagenomic analysis and mathematical modeling helped identify *Clostridium scindens*, while the stronger claim came from linking that organism to bile acid metabolism and experimental protection [2]. In personalized nutrition, a machine-learning model predicted postprandial glycemic responses, and the claim was strengthened by independent cohort evaluation and a blinded dietary intervention [3].

Microbiome science is vulnerable to this slippage because it often relies on indirect measurements. Stool can proxy for host-microbial interaction, taxonomic abundance for function, and inferred pathways for active biology. Each can inform interpretation, but each can also obscure the distance between signal and mechanism. Multi-omics narrows that distance only when a layer resolves a specific biological uncertainty.

## The multi-omics fallacy

The multi-omics fallacy is not the use of multiple molecular layers or a rejection of computational integration. It is claim inflation that occurs when cross-layer agreement is treated as mechanistic progress without a corresponding gain in measurement, localization, temporal resolution, functional linkage, or perturbation. Multi-omics can resemble biological completeness, and computational models can link uneven layers, detect latent structure, prioritize features, and generate plausible patterns that are useful for discovery but easy to overread as mechanism [1].

The boundary depends on the research aim. Exploratory studies can identify structure and prioritize candidates without turning coherence into explanation. Diagnostic models can support classification, prognosis, monitoring, or stratification without making predictive performance a marker of biological validity. Mechanistic studies can connect organisms, molecules, host responses, compartments, timing, and function, but inferred pathways, feature attribution, and cross-layer agreement are not causal architecture until the relevant biological links have been measured or tested.

Disease status is not a single biological variable. It compresses diet, medication use, inflammation, intestinal transit, stool consistency, immune tone, tissue turnover, clinical behavior, and sampling context into one analytic label. These variables can reshape microbial composition, gene expression, metabolites, host transcripts, immune markers, and clinical covariates. A multi-omics model may therefore reconstruct what disease does to a system without distinguishing causes from responses, adaptations, treatment effects, or collateral disruption.

The central problem is what the data are being asked to prove. A pattern that appears across several omics layers may be more interesting than a single-layer pattern, but repetition across layers does not make it causal. An added layer strengthens interpretation only when it reduces confounding, converts inference into measurement, establishes temporal order, localizes the signal, demonstrates host exposure, links a feature to activity, or shows that perturbing the proposed process changes the outcome.

This distinction is familiar from classical biochemistry, where independent experiments are used to resolve specific mechanistic uncertainties rather than restate an association in another form. Co-immunoprecipitation between an enzyme and substrate may support physical association, but stronger inference requires a different kind of test, such as showing that loss of the enzyme reduces formation of the predicted product. The evidentiary gain comes not from adding another measurement, but from answering a different mechanistic question. This distinction aligns with recent calls for microbiome research to emphasize credible inference, reproducibility, relevance, and causal restraint as artificial intelligence and machine-learning methods become more common in the field [1].

## Where multi-omics inference remains essential

Multi-omics inference should not be limited to studies that can immediately perform mechanistic validation. In large cohorts, rare disease studies, pediatric samples, archived specimens, low-volume tissue samples, and ethically constrained human studies, computational integration may be the only feasible way to identify structure, prioritize mechanisms, improve stratification, reveal cohort-specific dependencies, and guide the next measurement.

The boundary is not whether computational inference is used, but whether the claim is framed as association, prediction, prioritization, triangulation, biological plausibility, or verified mechanism. Longitudinal inflammatory bowel disease studies show how multi-omics can narrow uncertainty when design expands temporal and compartmental resolution. In the Integrative Human Microbiome Project, 132 subjects were followed for 1 year using integrated host and microbial profiles from stool, biopsy, and blood specimens [4].

## Assumption laundering

Assumption laundering occurs when an uncertain premise enters a computational workflow as a practical shortcut and exits as an apparent finding. It can occur when an inferred pathway is discussed as active metabolism, an imputed taxon as an observed organism, a predicted metabolite as host exposure, a stool signature as a mucosal mechanism, or feature attribution as causal biology. Table 1 maps common workflow points where assumptions can become stronger biological interpretations, along with safeguards that keep each output within its evidentiary lane.

**Table 1:** Common routes of assumption laundering in computational microbiome workflows.

Workflow stage	Practical assumption	Laundered interpretation	Safeguard
Sampling	Stool, saliva, swab, blood, or bulk tissue represents the relevant host-microbial interface	A sampled community is described as a mucosal, epithelial, immune, or tissue-level mechanism	State compartment limits and validate in the relevant site when mechanism is claimed
Sequencing	Reads represent true microbial signal	Contamination, host-read misassignment, or batch structure is interpreted as microbial biology	Use negative controls, contamination assessment, host-read filtering, batch evaluation, and sensitivity analyses
Annotation	Database assignment captures biological identity or function	Annotated genes or pathways are treated as known active functions	Report database limits and distinguish gene potential from activity
Imputation	Missing features can be statistically estimated	Imputed organisms, genes, or metabolites are discussed as observed	Label imputed features explicitly and avoid measurement language
Pathway prediction	Gene content implies pathway activity	Predicted pathways are described as active metabolism	Require transcript, protein, metabolite, flux, or functional evidence for activity claims
Metabolite prediction	Predicted metabolites imply host exposure	A predicted or fecal metabolite is treated as host exposure or immune signaling	Measure metabolites and establish compartmental relevance where exposure is claimed
Multi-omics integration	Cross-layer agreement implies directionality	Coherence across layers is written as a causal pathway	Require temporal order, confounder assessment, and perturbation where possible
Feature attribution	Model salience identifies biological importance	A model-important feature is described as a causal or host-relevant feature	Treat attribution as hypothesis generation unless independently tested
Interpretation	Prediction explains biology	A classifier is presented as mechanism	Separate diagnostic performance from biological interpretation

Prediction, imputation, attribution, and integration are legitimate when each output remains within its evidentiary lane. Prediction can classify, forecast, or stratify, but it does not verify mechanism. Imputation can support exploratory modeling, but it does not turn missing data into observation. Attribution can show what influenced a model, but not what caused a biological outcome. Integration can organize relationships across layers, but it does not prove causal architecture [5].

Prediction is valuable, but it is not biological explanation. A stool-based model that predicts colorectal cancer status does not show whether microbial features caused tumor biology, responded to it, or accompanied it. A measured fecal metabolite alone is not evidence of host exposure or functional effect.

## When computational confidence amplifies artifact

The cancer microbiome controversy shows how computational structure can become diagnostic or mechanistic biology before its foundations are secure. Low-biomass microbiome studies require special caution because contamination and cross-contamination can disproportionately affect samples near the limits of detection [6]. Poore *et al*. reported microbial signatures in blood and tissue across 33 cancer types and proposed diagnostic utility [7]. Gihawi et al. later concluded that data-processing errors, false-positive microbial assignments, and transformation-related artifacts allowed machine-learning models to generate apparently accurate classifiers [8]. The original article was later retracted by *Nature* [9]. The lesson is not that low-biomass microbial signals should be disregarded. It is that models can learn reproducible artifacts when disease labels are entangled with contamination, batch, reference-database error, normalization behavior, medication exposure, or sampling context.

The same concern applies to diagnostic microbiome models. A systematic review of 102 human gut microbiome classification studies found that only 12% reported Area Under the Curve using a genuinely independent test dataset [10]. A later review of 100 supervised machine-learning microbiomics studies found frequent limitations, including small sample sizes, incomplete demographic reporting, limited code availability, and inadequate reporting of independent testing or data leakage [11]. Reported performance can therefore appear more mature than the external evaluation beneath it.

Model bias and fairness belong in the same evidentiary frame. Microbiome datasets are often uneven across geography, ancestry, diet, medication exposure, socioeconomic context, sampling access, and sequencing practice. Publicly available human gut microbiome datasets remain strongly biased toward Europe and North America [12]. Gene and protein annotation resources are also biased toward well-annotated genes, which can steer pathway analysis, feature interpretation, and model priors toward biology that is already easier to see [13].

## Model interpretability and cross-layer coherence are not mechanism

Model interpretability and feature attribution methods can make model behavior more visible, but visibility is not mechanism. These methods can show which features helped a model reach an answer. They do not show that those features caused the phenotype, operated in the relevant tissue, preceded the outcome, or would change the outcome if perturbed. Interpretability remains a broad and unstable concept, spanning transparency, post-hoc explanation, trust, causality, and transferability rather than one clearly defined evidentiary standard [5].

The risk is that model explanations are easily mistaken for biological explanations. A highly ranked taxon, predicted pathway, or latent factor can justify a hypothesis, but it does not establish disease biology, altered metabolism, or an ecological state without independent evidence. Interpretability clarifies how the model organized the data, not what was biologically proven.

Cross-layer coherence creates the same risk in a different form by converting agreement into explanation. When microbial, metabolic, host transcriptional, and immune signals align, several biological layers may appear to tell the same mechanistic story. Agreement across layers still does not establish directionality. In observational systems, aligned signals can reflect shared confounding, disease severity, treatment history, sampling context, or downstream response rather than causal architecture.

Autism microbiome research illustrates the problem. Autism-related dietary preferences have been reported to mediate autism-gut microbiome associations, supporting a model in which dietary restriction and stool consistency could explain microbial differences otherwise vulnerable to interpretation as microbiome-driven neurodevelopmental biology [14]. This does not make the microbiome irrelevant to neurodevelopment. It shows that microbial associations may sit downstream of the phenotype they are used to explain. In multi-omics studies, that downstream structure can look more persuasive because several biological layers align. The alignment may be real while the causal interpretation remains unproven.

## From model output to biological claim

The field does not need a requirement that every computational study become a mechanistic study. Discovery, prediction, mechanism, and translation are different aims. The problem arises when claims outrun the evidentiary level reached. A model can detect structure, prioritize hypotheses, or support stratification without demonstrating why the structure exists or whether it operates in the host. The standard should be claim calibration rather than universal mechanistic validation.

A useful burden of proof states what each computational output has actually established. Computational signals should be evaluated for independent testing, cohort variation, pipeline sensitivity, batch structure, negative controls, and plausible confounders. The evidentiary status of each core feature should be named explicitly as measured, inferred, imputed, predicted, attributed, simulated, localized, perturbed, or clinically evaluated. Mechanistic claims require temporal fit, compartmental relevance, functional linkage, and, where possible, perturbation. Diagnostic or translational claims require evidence that the model improves diagnosis, prognosis, monitoring, stratification, or treatment selection beyond existing clinical variables. Table 2 translates this burden of proof into claim-calibrated standards for exploratory analyses, diagnostic model development, and mechanistic studies.

**Table 2:** Claim-calibrated standards for computational microbiome studies.

Study type	Appropriate claims	Minimum standards	Claims to avoid without added evidence
Exploratory analysis	Association, pattern detection, candidate prioritization, hypothesis generation	Transparent preprocessing, metadata completeness, confounder assessment, sensitivity analyses, pipeline robustness, and independent replication where possible	Causal, disease-driving, host-response, pathway-activation, or restoration claims
Diagnostic model development	Classification, risk prediction, prognosis, monitoring, treatment stratification	Train-test separation, leakage assessment, external evaluation, calibration, comparison with clinical covariates, decision relevance, and subgroup performance	Biological validity or mechanism based on model performance alone
Mechanistic dissection	Directional host-microbial pathway, causal mediator, functional mechanism	Direct measurement of key nodes, temporal order, relevant tissue or compartment, functional readout, and perturbation or rescue where possible	Active pathway, host exposure, causal mechanism, or therapeutic target from inferred or aligned features alone

These standards complement existing reporting and appraisal efforts rather than replacing them. Human microbiome studies should report design, sampling, laboratory, bioinformatic, and statistical details with sufficient transparency for interpretation and comparison, consistent with STORMS reporting guidance [15]. Diagnostic and prognostic microbiome models should also follow prediction-model standards, including TRIPOD+AI for complete reporting and PROBAST+AI for risk-of-bias and applicability assessment [16,17]. These frameworks do not determine whether a microbiome claim is mechanistic, but they help establish whether the computational evidence can support the claim being made.

Major biological conclusions should explicitly identify whether core features are measured, inferred, imputed, predicted, attributed, localized, perturbed, or clinically evaluated. Predictive performance should be separated from biological interpretation. A model may be clinically useful before its mechanism is known, and a mechanistic hypothesis may be biologically plausible before it is clinically useful.

Multi-omics integration should therefore be framed around the uncertainty each layer resolves, not the number of layers included. Feature attribution and interpretability methods should be used to identify which features require biological follow-up, not to convert model salience into mechanism. External evaluation should be treated as an evidentiary threshold for diagnostic claims, especially given repeated concerns about test-set discipline, data leakage, small sample sizes, demographic reporting, code availability, and generalizability in microbiome machine-learning studies [10,11].

The goal is not to make computational microbiome science less ambitious. It is to make it more useful. Computational models may be most powerful when they identify unstable features, reveal cohort-specific dependencies, quantify uncertainty, prioritize validation experiments, and show where additional measurement is needed. A computationally suggested feature should be judged by robustness, specificity, evidentiary status, and actionability. Robustness asks whether the signal persists across independent cohorts, reasonable preprocessing choices, pipeline perturbations, and negative controls. Specificity asks whether the signal is explained by site, batch, medication exposure, diet, stool consistency, disease severity, demographic structure, or other plausible nuisance variables. Evidentiary status asks whether the feature was measured, inferred, imputed, predicted, attributed, or simulated. Actionability asks whether the output identifies a concrete next measurement, validation experiment, localization test, perturbation, or clinical decision. The strongest role of computational modeling is not replacing mechanism, but clarifying which mechanisms are worth testing and which claims are not yet ready to be made.

## Conclusion

Multi-omics integration and computational modeling are central tools for finding structure, linking biological scales, and prioritizing experiments. Their power depends on keeping computation in evidentiary register. A model output is not a measurement. A prediction is not a mechanism. A coherent pattern is not biological resolution.

The multi-omics fallacy is not a rejection of biological integration. It is a warning that integrated molecular evidence can be overread when cross-layer coherence is treated as causal explanation. Artificial intelligence and machine-learning methods can intensify that risk when they give integrated associations the appearance of predictive precision, interpretability, or mechanistic completeness before the underlying biology has been established. Microbiome science has already spent decades learning that composition is not function, association is not causation, and stool is not the whole gut. Computational models can help correct those errors, or they can reproduce them faster, cleaner, and with greater authority.

The standard should be ambitious rather than defensive. Computational models should be used to expose uncertainty, identify fragile claims, reveal cohort-specific dependencies, improve study design, and direct experiments toward the mechanisms most worth testing. Mechanistic claims should be earned through measurement, localization, temporal order, functional linkage, and perturbation where possible. The future of computational microbiome science should not be models standing in for mechanisms. It should be models disciplined by biology, and mechanisms earned by evidence.
